# A qualitative analysis of health information seeking among African American adults in South Carolina

**DOI:** 10.1080/29944694.2025.2488107

**Published:** 2025-04-10

**Authors:** Sarah B. Maness, Jonathan H. Low

**Affiliations:** a Department of Health and Human Performance, College of Charleston, Charleston, South Carolina, USA; b School of Medicine, Meharry Medical College, Nashville, Tennessee, USA

**Keywords:** Health literacy, Minority Health, Social Determinants of Health, Health Inequity

## Abstract

Structural racism impacts education, economics, insurance coverage, and overall health. As a result, African American adults often have poorer access to quality healthcare, which is interconnected to social determinants of health. This study used a health and financial literacy approach to qualitatively determine how African American Adults in South Carolina find out about health information, how they decide the information is true, and how to pay for it (*n* = 20). Results indicated that participants place great value on the opinions of family and friends as well as health professionals and online information when seeking health information. These findings also appeared when examining how participants find out whether a piece of information is trustworthy or not. Additionally, participants reported difficulty navigating the healthcare system. This research identifies a need for both coordinated health literacy programming and structural changes in access to healthcare information in order to improve health literacy and access to healthcare among African American adults.

## Introduction

Structural racism impacts education, economics, insurance coverage, and overall health (Johnson-Agbakwu et al., 2022). As a result, African American adults often have poorer access to quality healthcare, which is interconnected to social determinants of health. This has a negative impact on health outcomes and is also reflected in higher healthcare costs (Weekes, 2012). South Carolina is ranked 44^th^ out of the 50 states in overall health and is affected by high poverty. Additionally, there are high rates of communities that do not have proper access to health care as well as an above-average population with undiagnosed and untreated chronic diseases (Health Tri-County, 2017). African Americans in South Carolina make up a high number of the population experiencing poverty and are disproportionately impacted by health issues (Merrell et al., 2020). In order to shed light on racial and ethnic health inequities to further enhance health equity among African Americans, this study identifies how African American adults seek health information and the challenges they face. The objectives of this study were to explore how African American adults in South Carolina 1) find health information, 2) decide if health information is true, and 3) find information on how to pay for healthcare.

## Methods

This study is part of a larger research study on financial and health literacy. This research contains information about health information-seeking. Study data on access to healthcare has been published elsewhere (Maness et al., 2023). Study methods are presented using the Consolidated Criteria for Reporting Qualitative Studies (COREQ) (Tong et al., 2007).

### Research team and reflexivity

Undergraduate and graduate research assistants (male and female) conducted all interviews. The principal investigator (SM, female) trained all interviewers prior to study initiation. To address researcher positionality, the principal investigator is a white female with a PhD. The other researchers identify with a range of racial/ethnic identities and study at either a regional small liberal arts college or a Historically Black College or University (HBCU). Interviewers did not have a relationship established with participants prior to study commencement. Participants did not receive prior information about the researcher beyond the goals of the study in the informed consent, and no characteristics were reported about interviewers (e.g. bias, assumptions, interests in the research topic).

### Study design

This study utilized thematic analysis as a methodological orientation. An interview guide was created by the study team members (SM, JL). The interview guide content analyzed for this manuscript includes the following questions:

“Next, I want to ask you some questions about where you hear about healthcare and where you would go to find out more information.

How do you find out about things important for your health?

- Prompts: friends and family, TV, radio, magazines, doctor (GP), nurse, other

How do you decide if the health information you find is true?

- Prompts: comes from trusted source? (if so, what/who is the source?), check with trusted source? (if so, what/who is the source?)

How do you find out about how to pay for health care?

- Prompts: Seeking information about health insurance? Cost of doctors’ visits or procedures?”

Participants (*n* = 20) were recruited online by study staff using purposive sampling through paid Facebook ads and online social media posts. Interested participants emailed study staff and were scheduled for a 30-min online qualitative interview. Interviews were conducted one-on-one between the participant and interviewer with no others present. Interviews took place from February to May 2022, were held on Zoom and auto-recorded and then further transcribed. This study did not utilize member checking of transcripts with participants. Participants also completed a voluntary online demographic survey using Qualtrics. Demographic questions included race/ethnicity, educational attainment, relationship status, work status, household income, county of residence, insurance coverage, type of insurance coverage, and time without health insurance in the last 12 months. Data collection continued until the study team determined that new insights were not being added and saturation was reached.

### Analyses

Qualitative data from the interviews were coded by two research team members (JL, TV) using thematic analysis according to Braun and Clarke (2006). Team members created a codebook, coded all transcripts, and reviewed the coding with the principal investigator (SM). Themes were not identified in advance but were derived from coding and analyzing the data. Study procedures were approved by the College of Charleston Institutional Review Board.

## Results

The voluntary survey was completed by 19 out of the 20 participants. 13 participants identified as female and six as males. All participants reported earning a degree higher than high school graduation and the total combined income of those living in their house during the past 12 months ranges between $10,000 and $90,000. More than half of the participants (*n* = 13) are employed, and the rest reported other work statuses such as going to school, retired, or being unable to work for health or family reasons. All the participants reported having health insurance. Thirteen participants reported private health insurance, three participants receive either Medicare or Medicaid and another three participants have health insurance that is provided by the military. Even though all participants had health insurance at the time of the demographic survey, there are three participants that stated, that there was a period in the last 12 months when they did not have any health insurance coverage.

### Theme 1: Finding out about things important to health through health professionals, family, friends, and online

Twelve out of the 20 interviewees stated that they seek health information through health professionals such as doctors, nurses, or specialists. This is based on trust in healthcare workers and satisfaction with how they interact with their patients (e.g. Interview 9, ll. 129–132). Besides consulting health professionals, there is also a strong pattern of African American Adults seeking health information through their close family and friends (*n* = 9). This is a result of the desire to avoid high healthcare expenses, as well as having a high level of trust in the community (e.g. Interview 10, ll. 109–114). Talking to family and friends is often seen as the first point of contact when it comes to health issues and questions. Getting assurance from family and friends in health-related topics before consulting a health professional stood out while interviewing the participants. This is reflected by one of the participants as follows:

Maybe talk to some friends or family that have gone through it, then once I am making an appointment with the doctor or nurse and I would talk with them. But I also have the ability to send email messages to my primary physician. So, I can ask them through email about issues that I have so that when I do come to the appointment, we are all on the same page in terms of what is going on and what kind of care I might need

(Interview 15, ll. 59–63).

Another source in which a theme was discovered because respondents mentioned it as a way to obtain health information is through work (*n* = 4). This is illustrated in the following example:

I definitely use my co-workers all the time. I ask, so where do you all go? Who have you been going to? What are your thoughts? … Everyone at work has been like here’s my dermatologist and here is who I think you should see, here is my favorite person, let me see if I can talk to them for you, that sort of deal

(Interview 7, ll. 204–208).

This theme results from the participants having trust in the opinions of their co-workers. However, three out of the four participants who talked about this specific way of health information-seeking are employed in a medical field and have health professionals at work that they can consult.

Six participants mentioned that they would Google health conditions, symptoms, and how to treat them or even look up reviews of different practitioners (e.g., Interview 3, ll. 100–104). It becomes apparent through those participants that they like to do their research online and on their own to get a better picture of the medical situation. However, the participants of the interviews also notice that not everything on the internet is true and some information might be misleading. This is elucidated by one of the participants as: *“You cannot believe everything people say, especially when it is coming from a Google or yelp review, regardless of how entertaining it is"* (Interview 7, ll. 223–225).

Another four participants get their health information through social media platforms such as Facebook or Instagram. Participants mentioned that they often use Facebook groups to connect with other people with the same or similar health conditions (e.g., Interview 3, ll. 100–102)

### Theme 2: Relying on research, health professionals, and family and friends to decide if health information is true

Nineteen out of 20 participants stated that they do further research after receiving medical advice, no matter what medium they got it from. The interviewees are making sure to get different opinions and investigate diverse sources to get a better overview and understanding of the situation or a health-related topic (e.g. Interview 16, ll. 167–168). More than half of the participants (*n* = 13) do their own research online to get a better picture of the situation. This may look something like this:

Most of the time, I just do research. To make sure of what is going on. I guess, to prove or to find out if what the source is telling me is the right thing. Well, usually Google. … I usually go online and do research like research for a paper or essay and try to use sources that are credible

(Interview 19, ll.134–139).

Some participants (*n* = 8) have even mentioned specific sources from which they obtain information and, more importantly, use them to double-check whether their health information is true. These sources are listed as follows:

- National Public Radio
- Local and National TV (Cable News Network, Microsoft and the National Broadcasting Company, and Fox News)
- Doctors/Hospital websites
- Centers for Disease Control and Prevention
- Government websites (.gov)
- Educational websites (.edu)
- Scientific articles/Peer-reviewed articles
- Medical forums
- Health Magazines
- Tricare
- Veterans Administration
- Medical Universities and Schools
- Pfizer Websites

This list consists of a variety of sources. Some of them are more reliable than others, but it is important to consider not just one but many sources when trying to seek health information and to distinguish whether a resource is trustworthy. Six out of the 20 participants attempted to do this, especially by staying away from sources that are not scientifically proven and can be put on the internet by anyone. This is well reflected by the following participant:

I try to stay away from Webmed. … I am trying to look at reputable websites, or I will go to a hospital website. There are universities like Vanderbilt and USC, like a medical university or a medical school. Then I try to go on their website to gain more information as well

(Interview 16, ll. 175–181).

An additional nine participants stated that they talk to their health professionals about health resources found from different mediums and discuss if they can apply them to their treatment plans (e.g., Interview 9, ll. 144–145). All of this is consistent with the participants’ engagement with their healthcare providers in Theme 1.

As further mentioned in the first theme, the participants have a strong bond with their family and friends, and they do trust their communities’ opinions a lot. This is also reflected in getting reassurance whether a health source might be true or not. Seven interviewees state that they talk to their family and friends in order to check if the health information received is trustworthy and whether they had similar medical experiences (e.g., Interview 18, ll. 96–97). This is emphasized once more by the following participant:

So normally what I would do is use multiple sources like, I say, talk to the nurses and doctors and I have friends that are physicians and things like that. So, I try to take in a lot of information and try to summarize it and get the best out of it and try to make a decision based on all the information I was able to get

(Interview 15, ll. 68–71)

### Theme 3: Hot to pay for healthcare using resources of health insurance and medical providers

When participants were asked about how they find out how to pay for healthcare, more than half of them (*n* = 12) stated that they find information through their health insurance providers. These participants make sure whether a certain doctor’s appointment or prescription would be covered by their health insurance and how much extra they would need to pay out of pocket (e.g. Interview 1, ll. 113–115). Participants state that they either look up this information on their insurance company’s website or app, receive emails with information in them, have a booklet they can check, or just call in and ask. An example follows:

That process is definitely through my health insurance. We do have an app through the health insurance, and then it lets you know if this doctor is in the network or out of the network. If it is a co-pay, etc. So, it is all pretty much just provided for us through the insurance company

(Interview 9, ll. 153–156).

An additional five participants stated that they will ask at the doctor’s office or the hospital how much the overall treatment or the co-pay will be before they considered going there (e.g., Interview 8, ll. 121–123). Only three participants stated that they would consider doing both, asking at the doctor’s office about how much it is and what payment options are available, as well as getting information through their health insurance companies before getting treatment. This supports the participants to get reassurance from both parties (e.g., Interview 16, ll. 194–198).

Four participants have also addressed the fact that navigating through healthcare is extremely hard these days, and it is often very confusing to understand. It is especially hard to understand what is covered by health insurance, how much the co-pay is, and what they actually end up paying for. It is further a challenge to grasp health-related terms to fully understand medical decisions and the associated costs. These participants feel overwhelmed by the pricing, lack of choices, and hidden surprises, where they end up having to pay more than originally expected (e.g., Interview 7, ll. 249–251). This is reflected by the following participant:

I think that there is a lot of deception in the medical field about pricing, but there is also a lack of choices. … I wish we all had more information to make a conscious decision about our health, but it is difficult. I feel like I am an intelligent person, but it is even hard for me to understand

(Interview 3, ll. 123–126).

## Discussion

The results of this study should be examined through the lens of the persistence of systemic racism and inequity in the United States, specifically in the American South. The participant sample in this study had a higher than average socioeconomic status, with all participants reporting health insurance coverage and some form of post-secondary education. It is important to acknowledge that this is not reflected across South Carolina nor nationally. Research indicates that racial inequities persist in uninsurance rates, with Black South Carolinians and Black Americans across the nation reporting higher rates of uninsurance than White or Asian Americans (SHADAC, 2022; CBPP, 2022). Historically, due to the lingering impacts of the racist practices of educational segregation and housing discrimination, it has been more difficult for African Americans to accumulate wealth, resulting in lower socioeconomic status (Herring & Henderson, 2016; Zucman, 2016). Discussion of the results must be interpreted with the acknowledgement that this sample is not representative of the larger population of African American adults.

Nevertheless, health information seeking is an area of importance to examine among populations of a wide range of socioeconomic status. While research indicates that people with high socioeconomic status are more likely to engage in health information-seeking behavior, they are also more likely to go online to diagnose themselves (Li et al., 2016). Research found no difference in trust of internet information based on educational attainment, which may present other risks in terms of the quality and accuracy of available information (Fareed et al., 2021).

Health information seeking is possible through various mediums, however, the most conventional way is through healthcare professionals (Miller et al., 2022). This is coherent with the results of the study since slightly more than half of the participants seek health information through their healthcare providers. From the statements of the interviewees, it can be concluded that the participants have the greatest confidence in the professional skills and knowledge of healthcare workers and seek their opinions as the main point of contact when health-related questions arise. The participants reported that they often turn to the doctor after they have discussed their health issues with family and friends. These results are interesting in light of our high socioeconomic status sample, in that previous quantitative research found that Black participants and patients with more education were less likely to use family, friends or coworkers as a source of health information (Jacobs et al., 2017). However, quantitative surveys may not be worded in a way that captures discussing health issues with family and friends and then following up with a doctor, introducing the need for a more nuanced understanding of pathways to seeking medical advice.

Additionally, it can be concluded that only a few individuals seek health information through work and their co-workers. This indicates a leakage of potential to promote health information through the workplace. Research shows that the workplace is the ideal place for public health officials to apply health promotion programs and to educate about healthy lifestyles since it is the ideal setting to reach a large number of people (Rongen et al., 2013). Hence, as only a few of the interviewees reported seeking health information through their work, a greater emphasis could be placed on promoting health through the workplace.

Many participants stated that they do their additional research online, which puts an even bigger emphasis on the importance of educating people on how to evaluate the accuracy of online information. Studies have shown that social media is a platform that enables patients to connect with people and allows them to have a social support network and a feeling of belonging, which can help them navigate illness (Pulido et al., 2020). However, the people sharing that information are often not health professionals and may share inaccurate information. People who are sick may be in a vulnerable situation where their opinion can be influenced by incorrect information (Tonsaker et al., 2014). The 21st century is shaped by easy access to information, limitless sources, and an abundance of data. Some of it is trustworthy and scientifically proven, but other sources should not be taken into consideration while seeking health information. This quantity of information makes it hard for people to navigate through the different mediums and to figure out which sources can be trusted (Tonsaker et al., 2014). Many participants stated that they discuss information that they previously found through a different medium with their health professionals. This can empower patients who want to play an active role in their care, and it also has a leveling effect on the relationship between patient and doctor. Discussing information found online with a doctor or a nurse can also have a positive effect on reducing anxiety (Bylund et al., 2009).

Regarding paying for health insurance, most participants stated they find out how to pay for healthcare by contacting their insurance company. This is a reflection of the finding that all participants are health-insured and have at least parts of their treatments covered. Participants also mentioned that they directly ask the hospital or the doctor’s office. This, however, can be difficult since often only an estimate of the price is given or medical staff are not familiar with the health insurance plans of their patients (Mulligan, 2017).

Finally, participants of this study stated that they have trouble navigating through the healthcare system. They mentioned that it is challenging to understand what their health insurance covers, what they have to pay on their own, and what they are specifically paying for. They discussed that they often have trouble understanding medical terms and medical decisions due to the complexity of terms and procedures. As a result of rapid growth and innovation in the healthcare system, it becomes difficult to navigate, and many people feel disoriented and stressed during the process (Griese et al., 2020).

This study is not without limitations. As an exploratory, qualitative study, it is not representative of the community. Our sampling strategy using social media may have introduced selection bias that resulted in our sample having a higher socioeconomic status and all participants being covered by health insurance. In order to see our recruitment materials, participants had to have internet access and a social media account, excluding those without internet access. As such, the limited socioeconomic diversity limits our ability to interpret findings of the health information seeking needs of other community members.

This research identifies a need for both coordinated health literacy efforts and structural changes in how to navigate the health insurance system to better serve African American communities and improve access to healthcare. Participants indicated frustration in understanding insurance and not being able to get all of the information from either the insurance provider or medical provider. Insurance companies, medical providers, and public health professionals must coordinate clearer and more accessible provision of accurate information about the type and pricing of medical care. Future research should include participants from a broad range of socioeconomic statuses and health insurance coverage. In order to tackle racial and ethnic health inequity, it is important to not only emphasize health communication and guidelines that help patients to fully understand the healthcare system but also the need for public services that make navigation of the healthcare system clearer and more accessible.
